# Incidental findings in MRI of the paranasal sinuses in adults: a population-based study (HUNT MRI)

**DOI:** 10.1186/1472-6815-14-13

**Published:** 2014-11-25

**Authors:** Aleksander Grande Hansen, Anne-Sofie Helvik, Ståle Nordgård, Vegard Bugten, Lars Jacob Stovner, Asta K Håberg, Mari Gårseth, Heidi Beate Eggesbø

**Affiliations:** Department of Ear, Nose and Throat, Head and Neck Surgery, St. Olavs Hospital NTNU, Trondheim, Norway; Department of Public Health and General Practice, NTNU, Trondheim, Norway; Department of Neuroscience, St. Olavs hospital NTNU, Trondheim, Norway; Department of Diagnostic Imaging, Levanger Hospital, Levanger, Norway; Department of Radiology and Nuclear Medicine, Oslo University Hospital, Oslo, Norway

**Keywords:** Incidental findings, Paranasal sinuses, MRI, Opacification, Mucosal thickening, Polyps, Retention cysts, Fluid, Population-based study

## Abstract

**Background:**

Diagnostic imaging of the head is used with increasing frequency, and often includes the paranasal sinuses, where incidental opacifications are found. To determine the clinical relevance of such findings can be challenging, and for the patient such incidental findings can give rise to concern if they are over-reported. Studies of incidental findings in the paranasal sinuses have been conducted mostly in patients referred for diagnostic imaging, hence the prevalence in the general population is not known. The purpose of this study was to determine the prevalence and size of incidental opacification in the paranasal sinuses in a non-selected adult population using magnetic resonance imaging (MRI) without medical indication, and to relate the results to sex and season.

**Methods:**

Randomly and independent of medical history, 982 participants (518 women) with a mean age of 58.5 years (range, 50–66) underwent MRI of the head as part of a large public health survey in Norway. The MRIs included 3D T1 weighted volume data and 2D axial T2 weighted image (WI). Opacifications, indicating mucosal thickenings, polyps, retention cysts, or fluid, were recorded if measuring more than 1 mm.

**Results:**

Opacifications were found in 66% of the participants. Mucosal thickenings were found in 49%, commonly in the maxillary sinuses (29%) where 25% had opacifications that were less than 4 mm in size. Other opacifications occurred in the anterior ethmoid (23%), posterior ethmoid (21%), frontal sinus (9%), and sphenoid (8%). Polyps and retention cysts were also found mainly in the maxillary sinuses in 32%. Fluid was observed in 6% of the MRIs. Mucosal thickening was observed more frequently in men than in women (*P* <0.05). No seasonal variation was found.

**Conclusions:**

In this large non-selected sample, incidental opacification in the paranasal sinuses was seen in two out of three participants, and mucosal thickening was seen in one out of two. Fluid was rare. Knowledge of incidental opacification is important because it can affect clinical practice.

## Background

Diagnostic imaging of the head and neck is used with increasing frequency [1] and often includes the paranasal sinuses where incidental opacifications, such as mucosal thickening, polyps, retention cysts, and fluid, are often found [2, 3], but the clinical relevance of these findings often remains uncertain for radiologists and ear, nose and throat surgeons. For the patient, such findings can cause unnecessary concern, and for the health system, they can potentially lead to unnecessary costs [4]. Different studies have used a variety of methods, and the findings in the paranasal sinus in both adult [2, 5–14] and paediatric populations [15, 16] have varied. In all studies on adults, the participants have been recruited from clinical settings, where the diagnostic imaging was performed primarily for diagnostic reasons [2, 5–8, 12, 17]. We therefore believe they cannot be considered a non-selected population. In previous studies the effect of sex and season on incidental findings has varied [3, 5, 12].

The HUNT study is a large public health survey in Nord-Trøndelag county in Norway that has been conducted in three waves between 1984 and 2009 (HUNT I, II and III). As a part of HUNT III, a random selection of persons between 50 and 65 years of age underwent an MRI of the head (the HUNT MRI study).

The purpose of this study was to estimate on MRI of a non-selected population, the prevalence and size of incidental opacifications of the paranasal sinuses, and to determine their relation to sex and seasonal variation.

## Methods

The Nord-Trøndelag health study (Helseundersøkelsen i Nord-Trøndelag, HUNT) is a large-scale epidemiological study conducted in three waves: HUNT I (1984 to 1986), HUNT II (1995 to 1997), and HUNT III (2007 to 2009), as a collaboration between the Norwegian Institute of Public Health, the Faculty of Medicine at the Norwegian University of Science and Technology, and Nord-Trøndelag County Council [18]. For each wave, the entire population aged 20 years or older and living in the Norwegian county of Nord-Trøndelag was invited to participate. Detailed questionnaires about health status, biomedical measurements, and blood samples were collected [18]. HUNT I resulted in 74,977 completed surveys, HUNT II resulted in 66,140, and in HUNT III resulted in 50,839.

From those participating in all three waves and who were aged between 50 and 65 years in HUNT III (n = 14,033), 1560 participants were randomly invited for an MRI study of the head [19]. Selection was made with no regard to health status. Exclusion criteria were travelling distance greater than 45 minutes to the MRI examination centre in Levanger and general MRI contraindications such as cochlear implants, severe claustrophobia, weight greater than 150 kg, cardiac pacemaker, or clipped cerebral aneurysm. Written informed consent was obtained from 1088 invitees (69%), and 82 of these did not come to the MRI examination [20]. In the period between July 21, 2007 and December 10, 2009, 1006 participants underwent an MRI. Of these, 21 were excluded due to artefacts, or if opacifications were seen on T1WI, but not fully demonstrated in T2WI (e.g. the base of the maxillary sinuses), and three due to extensive paranasal sinus surgery. Finally, 982 participants met all inclusion criteria (63% overall participation rate, 518 women, 464 men). Mean age was 58.5 years, age range 50–66 years (eight participants turned 66 years before the MRI had been done). The Regional Ethics Committee in Sør-Trøndelag, Norway approved the study (2011/2199-1).

For each participant, MRI was performed using a 1.5 T HDx scanner (Sigma, GE Healthcare, Waukesha, WI) equipped with an eight channel head coil and software version pre-14.0 M4. The scan protocol included axial T1 weighted images (WI), T1W magnetization prepared rapid acquisition gradient echo (MPRAGE) volume, scan axial T2WI, T2*WI and fluid attenuated inversion recovery (FLAIR) sequences, and a time of flight (TOF) 3D angio sequence. For this study we applied the axial T2WI (4 mm slices) and the T1W MPRAGE volume scan (1 mm slices). Scan parameters for the applied parameters are listed in Table 1.

**Table 1:** **MRI sequences and acquisition parameters**

MRI sequences	Matrix (pixels)	NSA	TR (ms)	TE (ms)	Flip-angle	Slice thickness (mm)	Gap (mm)	Overlap (mm)	FOV (mm)
**T1WI 3D GRE**	192 × 192	1	10	4	10°	1.2	0	0	240
**T2WI**	512 × 320	2	7840	95	90°	4.0	1.0	0	230

All imaging was performed on the same 1.5 T General Electric Sigma HDx 1.5 T magnetic resonance imaging (MRI) scanner equipped with an eight channel head coil and software version pre-14.0 M4. T1WI: T1 weighted image, GRE: gradient echo, T2WI: T2 weighted image, NSA: number of signal averages, TR: repetition time, TE: Time of echo, FOV: field of view.

Within two weeks after the MRIs were taken two experienced radiologists did a clinical evaluation of all MRIs in order to detect any pathology of the brain and the rest of the head with clinical significance for the participants. This evaluation was not particularly focused on sinus pathology. Three to four years later, in the period between April 2012 and July 2013, MRI readings and measurements of the sinuses were done independently and blinded for all participant data by an ear, nose and throat resident with 4 years’ experience (A.G.H), and a head and neck radiologist specialized in paranasal sinus radiology (H.B.E). A DICOM reader and associated software (Osirix version 3.2.4, 32 bit; Osirix Foundation, Geneva, Switzerland) were employed. In 21% of the cases there was a discrepancy in measurements or interpretation, and in these cases the MRIs were re-examined and a consensus was reached.

Each sinus was examined separately (i.e., left and right maxillary, anterior ethmoid, frontal, posterior ethmoid, and sphenoid sinuses) using both 3D T1W volume data with coronal, sagittal and axial reconstruction and 2D axial T2WI. Aplasia was recorded to calculate the prevalence of outcomes related to the number of sinuses. Frontal sinus aplasia [21] was defined as a lack of pneumatisation of the frontal bone with no ethmoid cells extending above a line tangential to the supraorbital margin, and sphenoid sinus aplasia [21] as pneumatisation limited to the pre-sphenoid bone.

Opacifications were categorized and defined as follows: i) Mucosal thickening, identified by a high signal on T2WI and a low signal on T1WI following the peripheral border of the sinus. ii) Polyps and retention cysts, identified as circumscribed, homogeneous, dome-shaped areas with high signals on T2WI. Polyps and retention cysts cannot be unambiguously differentiated by MRI [21], and were therefore merged in one group. iii) Fluid, identified on T2WI by a distinct air-fluid level, and measured from the sinus border to the air-fluid level. In each paranasal sinus, all opacifications were measured in millimetres (mm). The opacifications were visually determined at their maximum thickness, using both the T1WI and the T2WI. The opacifications visible, but measuring less than 1 mm were categorized as 0 mm. The superior walls of the paranasal sinuses are challenging to evaluate on axial images, and they were therefore mainly investigated on the coronal and sagittal T1WI. When mucosal thickening, polyps and retention cysts, and/or fluid were found in one and the same sinus, all opacifications were measured separately.

The sex and month of MRI was recorded. Seasons were defined as follows: April through October was categorized as summer, and November through March was categorized as winter.

### Statistical analysis

The prevalence and size of the three groups of opacifications were determined for all subjects and each sinus, and related to sex and season. The data were analysed using SPSS version 18 (released July 30, 2009). For comparison between men and women, and between seasons of MRI scan, the Mann–Whitney test was used for continuous variables (e.g. mean thickness of mucosal thickening, polyps/retention cysts, and fluid), and the Chi-squared test was used for proportions of participants with opacification (e.g. mucosal thickening yes/no, polyps/retention cysts yes/no, and fluid yes/no) for each sinus. In addition, prevalence of participants with opacifications in several sinuses was calculated. *P* ≤0.05 was considered statistically significant.

## Results

The prevalence of each group of opacification for each sinus, and for each sex is shown in Table 2. Frontal and sphenoid sinus aplasia was seen in respectively 49/982 (5%) and 1/982 (0.1%). Opacifications were observed more frequently in men (342/464, 73%) than in women (308/518, 59%), P <0.01. This was true both for mucosal thickening (men: 267/464, 57%, women: 17/518, 42%, P <0.01), and for polyps and retention cysts (men: 213/464, 46%, women: 166/518, 32%, P <0.01). Fluid was a rare finding, observed in one or several sinuses in 24/464 (5%) of the men and 32/518 (6%) of the women (P = 0.5). The majority occurred in the maxillary sinus.

**Table 2:** **Opacification defined as mucosal thickening, polyps, retention cysts and fluid level ≥1 mm in the right (R) and left (L) paranasal sinuses in 982 individuals (518 men and 464 women) on MRI**

Sinus	Maxillary		Anterior ethmoid		Frontal		Posterior ethmoid		Sphenoid	
	R	L	R	L	R	L	R	L	R	L
**Mucosal thickening ≥1 mm in each sinus, n and (** ***%*** **)**										
**Men**	154 *(33.2)*	147** *(31.2)*	125** *(26.9)*	125** *(26.9)*	47** *(10.1)*	57** *(8.8)*	115** *(24.7)*	129** (*27.8)*	49** *(10.5)*	37* *(7.9)*
**Women**	127 *(24.5)*	104 *(20.0)*	98 *(18.9)*	88 *(16.9)*	29 *(5.5)*	30 *(5.7)*	68 *(13.1)*	80 *(15.4)*	27 *(5.2)*	23 *(4.4)*
**Mean mucosal thickening in each sinus (mm ± SD)**										
**Men**	3.32 ± 3.61	3.52 ± 4.01	1.96 ± 1.09	2.06 ± 1.24	1.80 ± 1.32	1.54 ± 1.01	2.19 ± 1.29	2.14 ± 2.1	2.40 ± 2.70	2.20 ± 2.19
**Women**	2.55 ± 2.23	2.75 ± 1.78	1.96 ± 1.11	1.78 ± 1.29	2.17 ± 1.39	1.56 ± 0.86	1.95 ± 1.15	1.68 ± 0.9	2.70 ± 2.10	2.20 ± 1.52
**Polyps/retention cysts ≥1 mm in each sinus, n and (** ***%*** **)**										
**Men**	123** *(26.5)*	118** *(25.4)*	14 *(3.0)*	11 *(2.*3)	10 *(2.1)*	6 *(1.2)*	12 *(2.5)*	11 *(2.3)*	12 *(2.5)*	8 *(1.7)*
**Women**	85 (16.4)	79 *(15.2)*	8 *(1.5)*	8 *(1.5)*	1 *(0.02)*	5 *(0.1)*	9 *(1.7)*	7 (*1.3)*	12 *(2.3)*	6 *(1.1)*
**Mean polyp/retention cyst diameter in each sinus (mm ± SD)**										
**Men**	8.16 ± 5.17	9.14 ± 6.26	4.28 ± 2.33	3.81 ± 2.04	5.00 ± 1.88	5.83 ± 1.72	5.41 ± 2.42	3.63 ± 0.9	6.00 ± 4.19	6.87 ± 4.45
**Women**	8.24 ± 6.81	7.05 ± 4.44	2.12 ± 0.64	3.00 ± 0.92	-	4.00 ± 2.23	3.55 ± 1.23	6.42 ± 4.2	5.16 ± 2.08	5.33 ± 2.33
**Fluid ≥1 mm in each sinus, n (** ***%*** **)**										
**Men**	8 *(1.7)*	14 *(3.0)*	1 *(0.2)*	1 (*0.2)*	0	1 *(0.2)*	1 *(0.2)*	1 *(0.2)*	4 *(0.8)*	4 *(0.8)*
**Women**	12 *(2.3)*	16 *(3.0)*	1 *(0.2)*	0	0	0	0	0	5 (*1.0)*	5 *(1.0cps)*
**Mean fluid level in each sinus (in mm)**										
**Men**	6.6	7.5	-	-	-	-	-	-	7.3	5.3
**Women**	8.2	6.7	-	-	-	-	-	-	6.6	5.6

**p < 0.01, *p < 0.05 comparing men and women.

Number of sinuses is less than 982 due to aplasia in the right frontal (46/982, 6%), in the left frontal (46/982, 5%) and in the right sphenoid sinus (1/982, 0.1%). Mean calculated if n > 1.

**Figure 1:**
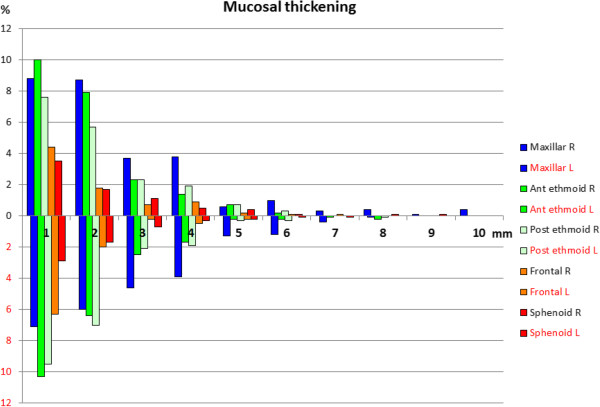
**Mucosal thickening.** Prevalence of mucosal thickening (in millimetres) with respect to each sinus and side, in 982 non-selected participants.

**Figure 2:**
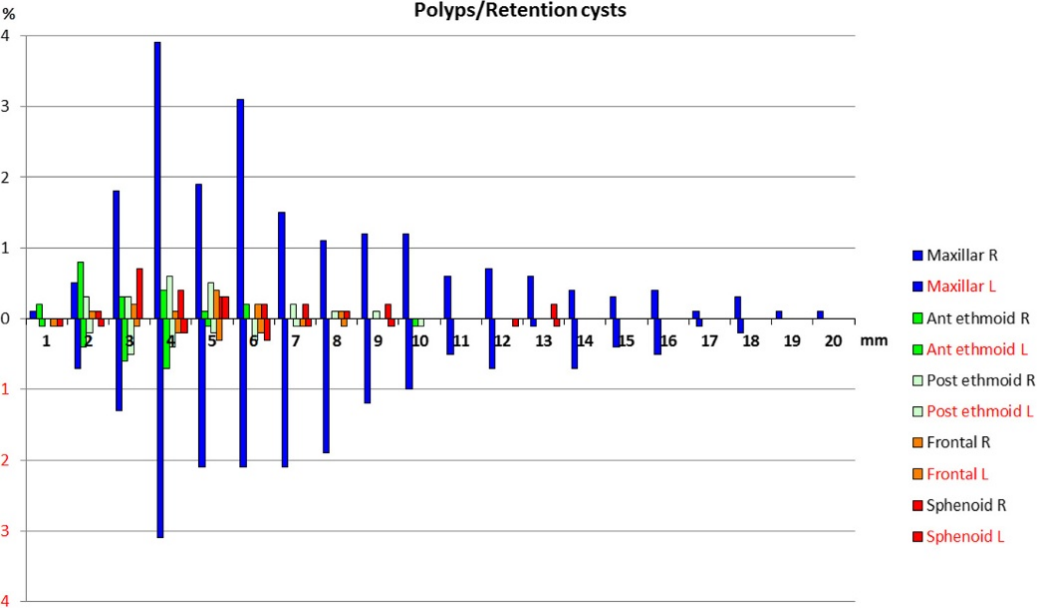
**Polyps and retention cysts.** Prevalence of polyps and retention cysts (in millimetres) with respect to each sinus and side, in 982 non-selected participants.

In both men and women, the highest prevalence of mucosal thickening was in the maxillary sinus, followed by the ethmoid (anterior and posterior) sinuses. For polyps and retention cysts, the great majority was found in the maxillary sinuses, and 317 of the 982 participants (32%) had either unilateral or bilateral polyps/retention cysts in the maxillary sinuses. Of these, 88 (9%) had this bilaterally. The proportion of mucosal thickening is demonstrated in Figure 1 and to the size of polyps and retention cysts in Figure 2. The results with alternative cut-offs for the mucosa (3 and 4 mm) are presented in Table 3.

**Table 3:** Prevalences (%) of paranasal sinus opacifications in 982 non-selected adult participants on MRI, using alternative cut-off values for mucosal thickening*

Cut-off	Maxillary	Anterior ethmoid	Posterior ethmoid	Frontal	Sphenoid
>3 mm*	26	4	4	2	3.5
>4 mm*	25	3	2.8	1.4	3.2

Prevalence of opacifications with respective cut-offs for mucosal thickening, and with polyps/retention cysts and fluid if >0 mm.

For each opacification group and each paranasal sinus, no seasonal variation was observed, except mucosal thickening in the left anterior ethmoid sinus, which was significantly more prevalent in MRIs taken during the summer (131/533 participants) than winter (82/449 participants), P = 0.01. Month-by-month comparisons revealed no significant variation.

## Discussion

To our knowledge, this is the first large MRI study reporting incidental findings in the paranasal sinuses in an adult, non-selected population, recruited for study purposes only. This is in contrast to previous studies [2, 5–8, 12, 17], where participants were examined for medical reasons. This study shows the prevalence and the range of mucosal thickening, polyps and retention cysts, and fluid in the different sinuses. Knowledge about opacifications of the paranasal sinuses in the general population is useful, because such findings are frequent, and can represent clinical challenges and give rise to costly additional investigations and unnecessary concern for the patient if interpreted wrongly.

It is a strength of the study that a detailed analysis of the participants has been published [19], showing that the HUNT MRI population was not considerably different from the general HUNT population. While the participants were non-selected in terms of health, they had somewhat higher education levels and were less likely to be overweight or have hypertension. There was no significant difference with regard to smoking, which is important for changes in the sinuses [22]. The fact that the study was not primarily aimed at investigating the paranasal sinuses makes participation bias unlikely (i.e. those with sinus problems are more likely to participate). Hence, we believe that the cohort is quite representative for the general Norwegian population of that age with regard to the parameters we have studied.

It is a limitation of the study that from the outset investigation the paranasal sinuses were not the main aim; hence MRI parameters may not be optimal for this purpose. All sinuses were completely visualized on 3D Volume T1WI with coronal, sagittal and axial reconstruction. If opacifications were seen on T1WI, but not covered by the T2WI (e.g. in the floor of the maxillary sinus), these participants were excluded. In addition, fluid can in some rear cases be present without a high signal on T2WI, or a distinct air-fluid level [23]. This can be a pus filled sinus, or allergic fungal sinusitis, and could have been missed with our definition of fluid. Although we hardly encountered such cases in this unselected material, the prevalence of opacification could be somewhat underestimated.

Also, the participants’ age did not represent the entire population; hence extrapolation of the results to other age groups must be done with caution. In addition, there were no data on sinonasal symptoms at the time of the MRIs, so we were unable to relate findings to current symptoms.

In the literature, reports on incidental sinus opacification vary, depending on patient selection and methodology used [5, 6, 8, 17]. Results have often been reported with pre-defined cut-offs, where definitions of a normal mucosal thickening ranges from <2 mm [13] to ≤4 mm [5]. Other studies have used the Lund-Mackay scoring system [14], modified Lund Mackay scoring systems [15], or have defined “minimal mucosal thickening” as normal without specifying a size range [17]. Still, there is no clear definition of normal or abnormal findings in the paranasal sinuses [5]. Furthermore, several studies only reported the most pronounced opacification in each sinus [5, 6]. This can potentially lead to an underestimation of findings, since one sinus may contain combinations of mucosal thickening, polyps, cysts, and fluid. In this study, we counted all visible opacifications that measured at least 1 mm, and there were no predefined cut-off values, which ensures that all findings that can give rise to problems in clinical practice are included.

In our study, the maxillary sinuses frequently showed thickening of mucosa (36% when both sides were considered together), and the majority of these were no larger than 4 mm, which accords with findings in other studies [2, 11, 12]. In Table 3, we present alternative cut off values and methods, as described by Gordts *et al.* [6] and Tarp *et al.* [5] for comparison of results. Gordts *et al.* [6] (MRI of n = 99), using a cut-off of >3 mm, found opacification in maxillary sinuses in 40% of the study participants. Similarly, Tarp *et al.* (MRI of n = 404), using a cut-off value of >4 mm, found a similar result (33.7%) [5]. Using the same cut-off values, our figures were lower, possibly reflecting that the other studies were performed in patients. In the other sinuses the differences were smaller (see Table 3).

Polyps and retention cysts were also more frequently seen in the maxillary sinuses (32%), which is in accordance with previous studies [2, 5, 11, 12, 24]. They were also of similar size and location as in the previous studies. In a comparable age group, Moon *et al.* [24] found a lower prevalence of polyps and retention cysts, but higher average cyst size (16–17 mm).

Tarp *et al.* [5] reported polyps and cysts in 15% of participants, the majority located in the maxillary sinuses. The higher frequency found in our study can be explained by the fact that we measured polyps and retention cysts even when opacifications of either of the other two groups were present, whereas Tarp *et al.* noted only the most pronounced abnormality.

Fluid was an infrequent finding in our study (6%), comparable to the findings by Gordts *et al.* (<5%) [11], Patel *et al.* (4%) [13], and Rak *et al.* (3%) [8].

Men had a significantly higher prevalence of mucosal thickening and polyps/retention cysts in this study. This is in agreement with most previous studies [5, 12, 25]. An exception is Maly *et al.* [7], who found opacification more frequently in women.

No clear seasonal variation was observed in this study. In a previous study, Tarp *et al.* [5] found a significantly higher degree of pathology during winter, whereas other studies have not shown significant seasonal variations [12, 25]. We chose the seasons mainly due to the climatic conditions in the area from which the participants were recruited, where the costal climate provides an early spring and late autumn. Nevertheless month-by-month comparisons did not show any significant variation.

## Conclusions

Our study shows that mucosal thickening, polyps, and retention cysts in the paranasal sinuses are frequent incidental findings on MRIs of the head in the general population. This study contributes to the knowledge of incidental findings in the paranasal sinuses due to its large participant sample from a general population. The results are important because opacifications in the paranasal sinuses challenges physicians and can have impact on clinical practice.

## Authors’ information

AGH is a resident in ENT and a research fellow.

ASH is RN and researcher.

SN is an otolaryngologist and professor.

VB is an otolaryngologist and associate professor.

LJS is a neurologist and professor.

AKH is a professor in medical imaging.

MG is a medical physicist.

HBE is a radiologist.

## Abbreviations

MRI: Magnetic resonance imaging
MPRAGE: Magnetization prepared rapid acquisition gradient echo
FLAIR: Fluid attenuated inversion recovery
TOF: Time of flight, mm: millimeter.
